# Loofah Sandwich Panels: The Effect of Adhesive Content on Mechanical and Physical Properties

**DOI:** 10.3390/ma15207129

**Published:** 2022-10-13

**Authors:** Robert Köhler, Marvin Jurisch, Aaron Kilian Mayer, Carsten Mai, Wolfgang Viöl

**Affiliations:** 1 Faculty of Engineering and Health, University of Applied Sciences and Arts, Von-Ossietzky-Straße 99, 37085 Göttingen, Germany; 2 Wood Biology and Wood Products, University of Göttingen, Büsgenweg 4, 37077 Göttingen, Germany

**Keywords:** *Luffa cylindrica*, loofah, sandwich panel, composite panel, adhesive content, polyester polyurethane elastomer

## Abstract

In the development of new materials, the focus nowadays is increasingly on their relevance with regard to lightweight construction or environmental compatibility. The idea of a lightweight sandwich panel was inspired by an increasing number of cosmetic accessories that use the fibers of the loofah plant, a rapidly renewable, light, fibrous raw material. The aim of the study was to develop a fiber composite panel based on the fibers of the loofah plant (*Luffa cylindrica*) as core material and wooden veneer as the skin layer to be used in areas of lead construction. Three different panel variations were produced for the tests, with a fiber–adhesive ratio between 1:1.05, 1:0.8, and 1:0.5. The mechanical strength (flexural strength and internal bond) and the physical properties (density and thickness swelling) were determined as a function of the fiber–adhesive composition. The results show that the flexural strength increased by approx. 400% and the thickness swelling was reduced by 10% with increasing adhesive quantity.

## 1. Introduction

Developments in the field of lightweight construction have increased steadily in recent years. For example, research is being conducted into lightweight materials to increase efficiency in the area of mobile transport [1,2]. In addition to lightweight construction materials such as fiber-reinforced plastics made from non-renewable raw materials, the use of renewable raw materials is becoming more and more important [3]. Sandwich panels are a useful approach to lightweight construction. Due to their high stiffness and low weight, they are used in a wide variety of applications. Sandwich panels based on paper, wood, and wood-based materials are becoming increasingly relevant.

Osei-Antwi et al. [4] produced sandwich panels using wood veneers as the face layer and cork as the core material. Britzke [5] described sandwich panels with a paper honeycomb core, which are used in furniture construction and in the manufacture of molded parts for vehicle interiors. Bartlome and Edgars et al. [6,7] showed wood-based sandwich panels in which wood fiber panels are joined together to form a sandwich structure.

Another possibility to produce a sandwich panel from renewable raw materials would be to use a natural fiber as core material, as it is used, e.g., in medium-density fibreboard (MDF) seaweed panels [8], which are planked with wood veneers. Such a plant that provides natural fibers is loofah (*Luffa cylindrica*), a Southern European/subtropical plant that has long been used as a natural sponge in many regions of the world [9]. The loofah plant belongs to the cucurbit family and is an annual crop. The size of the fruits varies on the plant location and ranges from 15 cm to 1 m, in some growing regions fruits over 1 m can also be harvested [10]. To obtain the fibers, the overripe fruits are harvested, dried, and then purified [11].

The chemical composition of the fibers is approximately 60% cellulose, 30% hemicelluloses, and 10% lignin, whereas the proportion can vary from 50–60%, 25–28%, and 10–12%. The different lignin and cellulose contents depend on environmental and metabolic factors [12]. Compared to wood, this offers the decisive advantage that the short period between sowing and harvesting can provide large quantities of fiber material on a regular basis [13]. The areas of application are numerous; in addition to its use as a household or cosmetic sponge, it is also used as a packaging additive [14]. In addition, the Luffa fibers are used in reinforced composites, as described [15,16]. Since the Luffa fibers, as determined in this study, have good interlocking properties in their chopped form and have a low material density of 870 kg/m^3^ compared to other natural fibers (hemp: 1480 kg/m^3^; cotton: 1550 kg/m^3^), they can be very well suited for use as the core material of a sandwich panel [17,18].

Adeyanju et al. [16] provided an overview of reinforced composites of Luffa fibers and different polymer composites. The most common polymers are epoxy resins, polyesters, polypropylene, polyethylene, and vinyl esters. Melo et al. [19] showed eco-composites, which were prepared from castor oil polyurethane reinforced with *Luffa cylindrica* fibers.

Furthermore, the use of polyurethane resins for MDF production leads to good mechanical properties such as internal bond strength and bending strength. Even relatively low resin contents exhibit mechanical properties, which surpass the requirements of European standards [20,21]. In addition, an adhesive was selected, which, according to its specification, is compostable [22].

The aim of this study is the production of fiber sandwich panels based on Luffa and polyester polyurethane resin in accordance with different binder contents. The feasibility of producing such a sandwich panel should be confirmed and the mechanical and physical properties determined.

## 2. Materials and Methods

**Figure 1:**
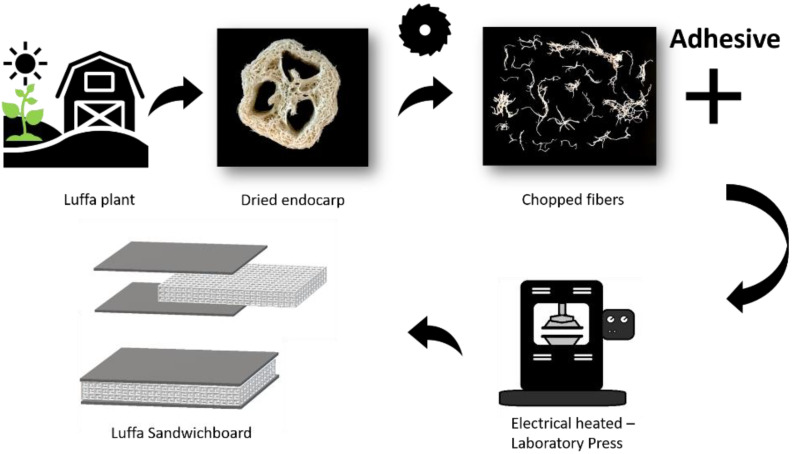
Schematic of the Luffa sandwich panel production.

The manufacturing process is shown in Figure 1 and is divided into five parts. The core material of the sandwich panels consists of the dried fibers of the Luffa plant, which come from production leftovers from the cosmetics industry (Ibérica de esponjas vegetales s.l.u, Pontevedra, Spain).

In order to obtain a homogeneous and fine fiber structure in the panel core, the Luffa fruits were shredded in a knife shredder (Güde GH 651 B, GÜDE GmbH & Co. KG, Wolpertshausen, Germany) to a fiber length between approx. 5 to 50 mm (measured with a ruler). The fibers were impregnated by submersion until fiber saturation with a water-based polyester polyurethane elastomer adhesive with a solids content of 40% (Epotal^®^ Eco 3702, BASF SE, Ludwigshafen, Germany) and then dried for 4 h at 40 °C. The Luffa fibers have a measured water absorbency of 264 ± 3%, which, when the adhesive is diluted to a solids content of 20%, results in a fiber-to-adhesive ratio (FAR) of 1:0.53 (type A). Furthermore, the adhesive was diluted to a solids content of 30%, resulting in an FAR of 1:0.79 (type B), and the undiluted adhesive resulted in an FAR of 1:1.05 (type C). All FAR are expressed as dry mass of fibers to dry mass of binder. In order to use the same amount of fiber and adhesive for all plates (starting from the absolute dry material mass), depending on the solids content of the adhesive, after the drying the moisture content of the fiber–adhesive mixture had to be determined, using a Moisture Analyzer (Sartorius MA, Sartorius Lab Instruments GmbH & Co. KG, Göttingen, Germany). Based on the measured value, which was approx. 33 ± 10%, the mass of the core material to be used could be calculated for each formulation before pressing. The boards were produced in a 2-step process. In the first process step, the fiber adhesive mixture was pressed to a constant thickness of 16 mm for 25 min at 150 °C using a hot press (Joos LAP 40, Gottfried Joos Maschinenfabrik GmbH & Co. KG, Pfalzgrafenweiler, Germany). After cooling down to 20 °C, in the second process step, adhesive-coated (81.6 ± 9.1 g/m^2^) (Epotal^®^ Eco 3702, BASF SE, Ludwigshafen, Germany) 1.5 mm thick peeled beech veneers were applied to the fiber core at 90° to each other and pressed again at 150 °C for 15 min, whereby the panel thickness was kept constant at 16 mm. To prevent delamination of the sheets, the sandwich panel was pressurized with 2 kPa after the pressing process and cooled down to 20 °C until the adhesive had completely cured.

For a better understanding of the produced sandwich panels, the density, as well as the cross-sectional density profile, was recorded using a Fagus-GreCon Density Profiler (Fagus-GreCon Greten GmbH & Co. KG, Alfeld, Germany) with an X-ray power of 23 kV and a feeding speed of 0.05 mm s^−1^. Eight specimens of each sample type with dimensions of 50 mm × 50 mm (L × W) were analyzed.

To determine the bending strength a three-point bending test was performed using an Instron 5567 Tensile and Compression Tester (Instron GmbH, Darmstadt, Germany) according to the European standard EN 310 [23]. Ten samples of 450 mm × 50 mm (L × W) of each formulation were produced and the modulus of rupture (MOR) and modulus of elasticity (MOE) were determined.

For the calculation of the MOR and the MOE the following equation was used:

(1) $$\mathrm{MOR}=\frac{3\;F_{max}\;l_{1}}{2\;b\;t^{2}},$$

(2) $$\mathrm{MOE}=\frac{l_{1}^{3}\;\left(F_{2}-F_{1}\right)}{4\;b\;t^{3}\;\left(a_{2}-a_{1}\right)},$$

with MOR modulus of rupture, *F_max_* breaking force, *l*_1_ distance between support points, *b* specimen width, *t* specimen thickness, MOE modulus of elasticity, *F*_2_ *− F*_1_ the increase in force in the rectilinear area of the force–deflection diagram and *a*_2_ *− a*_1_ the deflection increase in the specimen center (corresponding to *F*_2_ *− F*_1_).

The internal bound strength test was conducted to the European standard EN 319 [24] using an Instron 5567 Tensile and Compression Tester (Instron GmbH, Darmstadt, Germany). In accordance with the standard, five specimens of 50 mm × 50 mm (L × W) of each formulation were produced. For the calculation of the internal bond the following equation was used:

(3) $$IB=\frac{F_{max}}{a\cdot b},$$

with *IB* internal bond, *a* and *b* specimen length, and width.

The thickness swelling was analyzed in accordance with the European standard EN 317 [25]. Eight samples with dimensions of 50 mm × 50 mm (L × W) were determined whereas the samples were stored in water for 0.5, 2.5 h, 12 h, and 24 h, using an outside micrometer. The measurement was performed at 20 °C and 65% relative humidity

Before each test, the samples were conditioned to 20 °C at 65% relative humidity.

To ensure a normal distribution (α = 0.05), a Kolmogorov–Smirnov normality test was used. The data were analyzed with Tukey’s HSD (honestly significant difference) tests (α = 0.05) to identify statistical differences between the variants.

## 3. Results and Discussion

### 3.1. Density and Thickness

**Figure 2:**
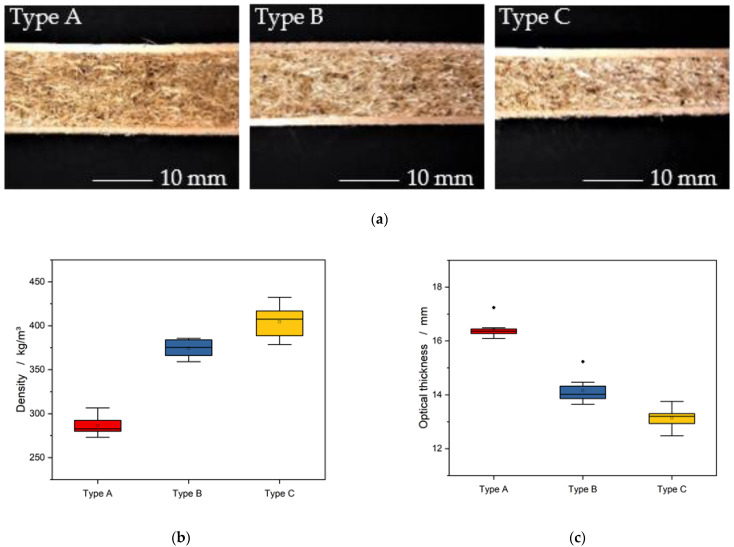
(**a**) Cross-section of the produced loofah sandwich panels of different types, with associated mean density (**b**) and panel thickness (**c**).

Figure 2a–c shows the cross-section, the average density, and the thickness of the Luffa sandwich panels variants. With an increase in the proportion of adhesive, the density increases from 286 kg/m^3^ (type A) to 374 kg/m^3^ (type B) and 404 kg/m^3^ (type C).

As the mass of the material used for the core in the sandwich panel remains the same and the proportion of solid material in the adhesive increases, there is less fiber material in the panel, which leads to a more compact structure and increases the overall density of the sandwich panel. At the same time, the resistance that counteracts the pressing forces during production also decreases. This resulted in panel thicknesses of 16.4 mm, 14.2 mm, and 13.1 mm for the FAR in type A, type B, and type C, respectively. Although all panels were pressed to 16 mm, there were different degrees of shrinkage of the panel thickness during the production process. This may be due to the high proportion of adhesive in the boards, which is embedded in the empty spaces between the fibers during the pressing process and thus hardly contributes to the total volume. The volume shrinkage of the panels could be directly related to the solidification of the polyester polyurethane adhesive [26,27]. Only the specimen with the lowest adhesive content was able to resist shrinkage and to maintain, respectively, relaxed beyond, the targeted panel thickness due to its high fiber content (type A).

**Figure 3:**
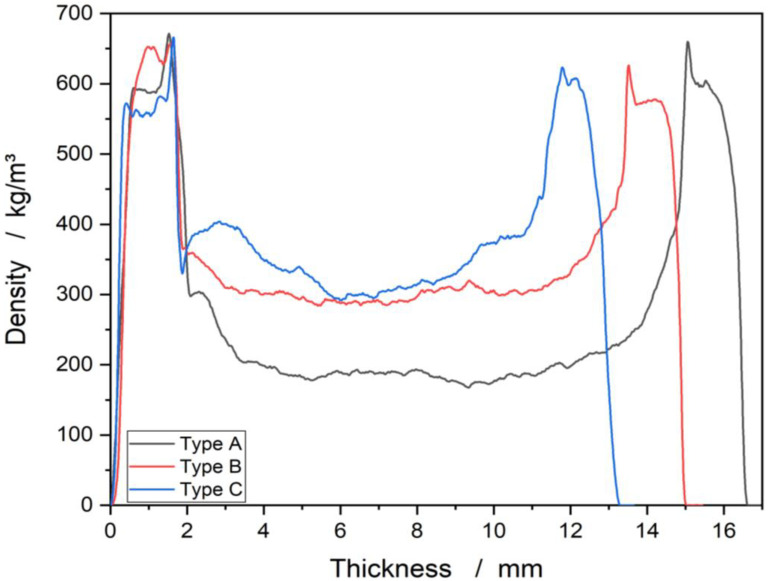
Density profile across the panel cross-section.

Figure 3 shows the density curve across the panel cross-section. The edge layers exhibit a density of 550–600 kg/m^3^ [28], which can be assigned to the beech veneers. It follows, at a thickness of 1.5–2 mm, an increase in density to a value of 650 kg/m^3^, which can be attributed to the adhesive layer [29]. As the profile progresses, the different densities of the individual types A, B, and C core materials can be differentiated from the edge layers [12]. The density profiles support the results of Figure 2a where type A has the lowest density followed by type B and type C. The lowest core density of about 200 kg/m^3^ is attributed to type A. Whereas type B and type C exhibit a higher core density. When comparing the density curves in Figure 2 and the raw densities in Figure 3, it can be observed that the edge layers and the beech veneers have a high influence on the total density.

### 3.2. Modulus of Rupture and Modulus of Elasticity

**Figure 4:**
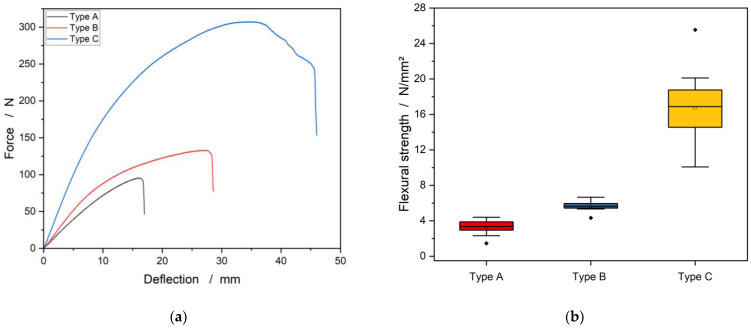
The influence of the FAR to: (**a**) load–deflection curves and (**b**) to the flexural strength of the Luffa sandwich panels.

Figure 4 shows the effects of the change in the FAR on load–deflection curves and the flexural strength in the three-point bending test. The load–deflection curves of the loofah sandwich panels displayed plastic deformation following the initial linear elastic region of the curve. As the proportion of FAR increases, a higher deflection and a higher fracture force are obtained (Figure 4a).

With the smallest fiber-to-adhesive ratio in the type A panel, maximum flexural strength of 3.3 N/mm^2^ was achieved on average (Figure 4b), with a modulus of elasticity of 276.1 N/mm^2^. During the tests, it was noticed that the amount of adhesive was not sufficient to transfer the forces inside the fiber core evenly to all fibers (comparable to FRP, see [30,31]) and to cross-link all fibers. This led to the splitting of the specimens, i.e., a fibrous rupture of the fiber core and delamination of the cover layers.

With panel type B having a maximum flexural strength of 5.6 N/mm^2^, a modulus of elasticity of 436.3 N/mm^2^ was achieved. With this type, the specimens behaved significantly tougher during the test than with the previously described variant. The fiber layer showed good cohesion and did not split during the test. It can also be seen that the top layer has significantly better adhesion to the core. The upper cover layer only broke abruptly when the maximum force was reached and detached from the fiber core.

The type C specimens, with a maximum flexural strength of 16.8 N/mm^2^, reached a modulus of elasticity of 727.9 N/mm^2^. During the bending test, these specimens showed similar behavior to the type B specimens, except that the bonding strength between the fibers in the core and between the fiber core and the cover layers was significantly higher. During the entire bending test, the upper cover layer only detached from the fiber core where the compression was at its maximum, while the rest of the specimen remained intact. The specimen proved to be particularly stiff and broke open abruptly when the maximum tension was reached at the bottom cover layer.

### 3.3. Internal Bond Strength

**Figure 5:**
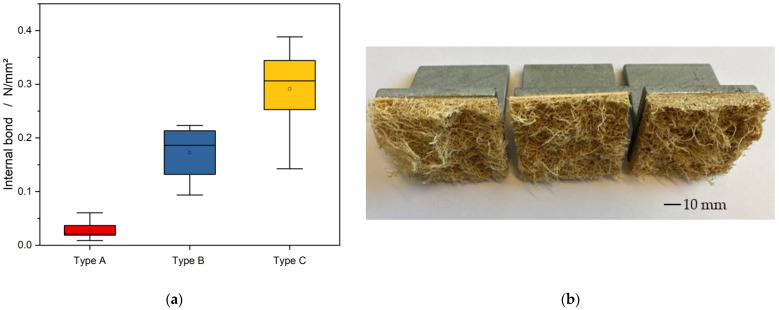
(**a**) The influence of the FAR to the internal bond strength of the Luffa sandwich panels. (**b**) Luffa sandwich panels after the internal bound strength test (from left to right Type A, Type B, and Type C).

Figure 5 shows the influence of the FAR on the IB which was calculated using Equitation (3). The sample performances were comparable to that of flexural strength. The sandwich panels of type A have an IB of 0.03 N/mm^2^. With an increasing proportion of adhesive in specimens B and C, compared to specimen A, the IB strength increases to 0.17 N/mm^2^ and to 0.29 N/mm^2^.

As with the flexure test, the specimens failed due to cracks in the core material, and delamination of the Type A formulation could be observed in individual cases. When the specimens were pulled apart, it could be seen that the fiber–adhesive bond had loosened, and the orientation of the fibers was in direction of tension (see Figure 5b). The reason for this could be the insufficient solid content of the adhesive, which leads to insufficient adhesion of the fiber mat. Compared to other fiber composite materials, it has also been observed that a lack of matrix material can lead to a weak structure [30].

Due to the higher proportion of adhesive in the panel composite, it is possible that the fibers were better anchored in the fiber–adhesive composite, which prevented the sandwich panel from splitting. Furthermore, it was found that an increasing proportion of adhesive counteracted delamination of the sandwich panel.

### 3.4. Thickness Swelling

**Figure 6:**
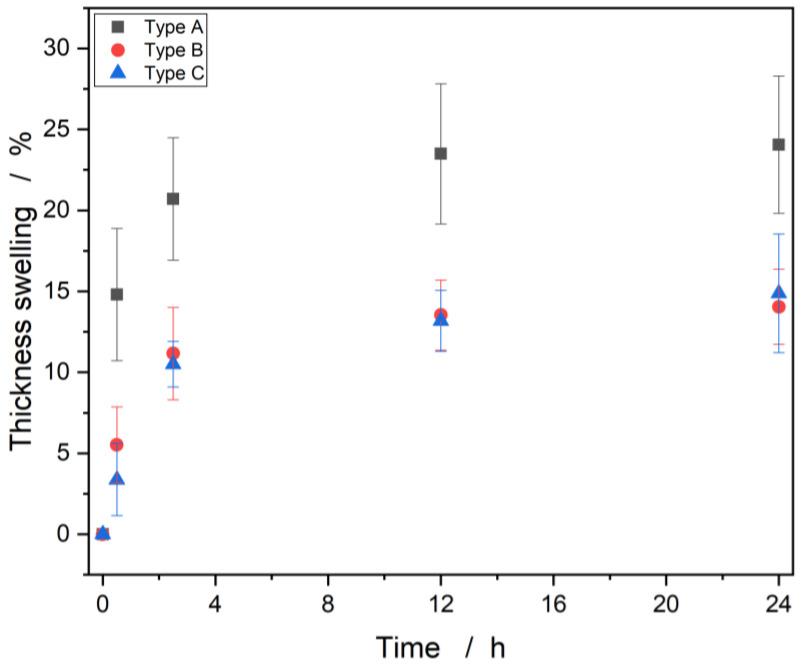
Thickness swelling as a function of time.

Figure 6 shows the swelling behavior of the samples as a function of time. The samples of panel type A, B and C showed a swelling of approx. 24% and 14% after 24 h. Thickness swelling occurred in the first 12 h and thereafter saturation was achieved for all panel types. The strong swelling of sample A is attributed to the largest proportion of Luffa fibers among the formulations. The fibers can absorb about 2.5 times their mass in water, which leads to a large increase in volume. The two types B and C exhibited a very similar thickness swelling, which suggests that even at a fiber-to-adhesive ratio of 1:0.8 (type B) there is enough adhesive to coat the fibers and protect them from swelling.

## 4. Comparison to DIN Standard for Fiber Panels

Based on the density values achieved, the sandwich panels can be compared with wood fiber panels. The comparable materials are porous boards/soft boards (SB) and medium–hard fiberboards with low density (MBL), which are defined in a range of less than 400 kg/m^3^ and between 400 and 560 kg/m^3^, respectively, see Table 1 [32,33].

The tension values of types A and B can be compared with the standardized values for SB fiberboards for general purposes in dry areas (0.8 N/mm^2^) or also SB.E for general purposes in outdoor areas (0.9 N/mm^2^). Type C is comparable to the limits of MBL for general purposes in dry conditions (8 N/mm^2^) or also to MBL.E which is intended for outdoor use (12 N/mm^2^) [34].

With regard to the internal bond strength, no comparison can be made with SB and MBL fiber materials, as the standard only includes values from a higher density range (MBH). MBH fiber boards have a specified internal bond strength of 0.3 N/mm^2^, this value is almost reached by board type C [34].

The comparative values for swelling can also be obtained from the DIN standard (DIN EN 622-3,4). For SB and MBL materials, a swelling of 6–10% or 9–15% is specified depending on the application [34].

## 5. Conclusions

In this study, three different Luffa sandwich panels with different adhesive–fiber ratios were produced. The results of the investigations allow the following conclusions to be made:

- Boards with densities of 286 kg/m^3^ to 404 kg/m^3^ could be produced which have a thickness of 13.1 mm to 16.4 mm.
- With increasing density:
  ○ The modulus of elasticity could be raised by 164%
  ○ The internal bond strength could be raised by 409%
- By increasing the adhesive–fiber ratio, the thickness swelling decreased by 10%

The Luffa sandwich panel could be used as an alternative (lightweight) construction panel and compares well with the SB and MBL-type fiber boards.

However, the thickness swelling and the bonding between the fiber core and the face layer is a problem in offering a reliable and competitive board material. Therefore, further investigations should be carried out to determine whether the volume shrinkage of the panels is directly correlated to the solidification of the polyester polyurethane adhesive. Even though the usage of the polyester polyurethane binder was feasible for the production of Luffa wood sandwich panels, the adhesive content has to be reduced in order to produce a board that is also attractive from an economic point of view [35].

## Figures and Tables

**Table 1:** Comparative figures.

Board Type	Thickness Swelling [%]	Flexural Strength [N/mm^2^]	Modulus of Elasticity [N/mm^2^]
MBL	9–15	8–12	-
SB	6–10	0.8–0.9	-
Type A	24.05	3.3	276.1
Type B	14.04	5.6	436.3
Type C	13.93	16.8	727.9

## Data Availability

Not applicable.
